# Targeted inhibition of the methyltransferase SETD8 synergizes with the Wee1 inhibitor adavosertib in restraining glioblastoma growth

**DOI:** 10.1038/s41419-023-06167-3

**Published:** 2023-09-27

**Authors:** Rosa Della Monica, Michela Buonaiuto, Mariella Cuomo, Cristina Pagano, Federica Trio, Davide Costabile, Giulia de Riso, Francesca Sveva Cicala, Maddalena Raia, Raduan Ahmed Franca, Marialaura Del Basso De Caro, Domenico Sorrentino, Giovanna Navarra, Laura Coppola, Lorella Tripodi, Lucio Pastore, Juergen Hench, Stephan Frank, Claudio Schonauer, Giuseppe Catapano, Maurizio Bifulco, Lorenzo Chiariotti, Roberta Visconti

**Affiliations:** 1 CEINGE-Advanced Biotechnologies “Franco Salvatore”, Napoli, Italy; 2 Department of Molecular Medicine and Medical Biotechnologies, University of Napoli “Federico II”, Napoli, Italy; 3 SEMM-European School of Molecular Medicine, University of Napoli “Federico II”, Napoli, Italy; 4 Pathology Unit, University of Napoli “Federico II”, Napoli, Italy; 5 Institute for Medical Genetics and Pathology, Basel University Hospitals, Basel, Switzerland; 6 Neurosurgery Unit, “Antonio Cardarelli” Hospital, Napoli, Italy; 7 Neurosurgery Unit, “Ospedale del Mare” Hospital, Napoli, Italy; 8 Institute for the Experimental Endocrinology and Oncology “G. Salvatore”, National Council of Research of Italy, Napoli, Italy

**Keywords:** Targeted therapies, Checkpoints

## Abstract

Despite intense research efforts, glioblastoma remains an incurable brain tumor with a dismal median survival time of 15 months. Thus, identifying new therapeutic targets is an urgent need. Here, we show that the lysine methyltransferase SETD8 is overexpressed in 50% of high-grade gliomas. The small molecule SETD8 inhibitor UNC0379, as well as siRNA-mediated inhibition of SETD8, blocked glioblastoma cell proliferation, by inducing DNA damage and activating cell cycle checkpoints. Specifically, in p53-proficient glioblastoma cells, SETD8 inhibition and DNA damage induced p21 accumulation and G1/S arrest whereas, in p53-deficient glioblastoma cells, DNA damage induced by SETD8 inhibition resulted in G2/M arrest mediated by Chk1 activation. Checkpoint abrogation, by the Wee1 kinase inhibitor adavosertib, induced glioblastoma cell lines and primary cells, DNA-damaged by UNC0379, to progress to mitosis where they died by mitotic catastrophe. Finally, UNC0379 and adavosertib synergized in restraining glioblastoma growth in a murine xenograft model, providing a strong rationale to further explore this novel pharmacological approach for adjuvant glioblastoma treatment.

## Introduction

Glioblastoma, a WHO grade 4 astrocytic tumor of the central nervous system, is the most malignant brain cancer. The treatment of choice remains, when feasible, maximal surgical resection followed by concurrent radio- and chemotherapy with temozolomide, but the prognosis remains poor [1]. Numerous clinical trials designed so far to individuate new, more effective treatments for glioblastomas have not led to significant improvements. Even the immune checkpoint inhibitors, a major breakthrough in the therapy of many cancers, have failed to improve glioblastoma prognosis [2]. The few drugs recently approved, such as larotrectinib and entrectinib for NTRK-fusion-positive glioblastomas, are all restricted to few patients and to second-line treatment for recurrent cancers [3]. Thus, the identification and validation of new therapeutic targets is crucial to improve the outcome of patients affected by glioblastoma.

Recently, it has been demonstrated that the lysine methyltransferase SETD8 is overexpressed in many solid and hematological human cancers, its higher level of expression being associated with a greater tumor aggressiveness [4]. Accordingly, SETD8 has been suggested as a promising therapeutic target for high-grade serous ovarian cancer [5].

SETD8 (also known as PR-Set7 or KMT5A), a member of a large family of lysine methyltransferases, catalyzes the mono-methylation of histone H4 on lysine 20, transcriptionally silencing chromatin [6]. SETD8-induced methylation of H4 is also a prerequisite for the recruitment of 53BP1 on chromatin, at double-strand break DNA damage sites, to finally and efficiently activate DNA repair [7]. Accordingly, depletion of SETD8 by siRNAs abrogates 53BP1 foci formation at DNA double-strand breaks and, in turn, DNA repair [7]. SETD8 methyltransferase activity also targets non-histonic proteins, many of them with cancer-driving functions. By methylating the Proliferating Cell Nuclear Antigen (PCNA) protein, SETD8 induces proliferation of numerous cancer cell lines [8]. Moreover, in neuroblastoma cells, SETD8 catalyzes the methylation on lysine 382 of p53, resulting in loss of the p53 tumor suppressive functions [9, 10]. Hence, SETD8 inhibition rescues p53 activity, arresting neuroblastoma cell cycle in G1/S [10]. SETD8 activity on p53 has also been investigated in myeloma cells. SETD8 inhibition results in cell cycle arrest in G1/S or in G2/M, in p53-proficient or -deficient myeloma cells, respectively [11]. To summarize current knowledge, many SETD8 functions appear to be cell-specific, reasonably a consequence of the diverse substrates methylated and because histone H4 methylation might have different impact on transcription, depending on the genomic- and/or cellular context [4].

Up to now, there is no data about SETD8 function in glioblastomas. Thus, we investigated SETD8 expression and activity in glioblastoma tissues, cell lines, and primary cells, demonstrating that SETD8 inhibition induces DNA damage and, in turn, cell cycle arrest at safeguard checkpoints. Most importantly, the inhibition of the cell cycle checkpoint kinase Wee1 permits SETD8-inhibited, DNA-damaged cells to bypass the checkpoints and to progress into mitosis where they die by mitotic catastrophe. Finally, we demonstrate the efficacy of SETD8+Wee1 targeted inhibition in a glioblastoma xenograft mouse model.

## Materials and methods

### Cell cultures and chemical treatments

U87MG, LN-18, and U251 cells were grown in DMEM (Sigma-Aldrich, St. Louis, MO, USA) with 2 mM L-glutamine (Sigma-Aldrich) and 10% fetal bovine serum (FBS; Thermo Fisher Scientific, Waltham, MA, USA). SW1088 cells and primary isolated glioblastoma cells were grown in RPMI (Sigma-Aldrich) with 10% FBS, 2 mM L-glutamine and 1% non-essential amino acids (Sigma-Aldrich). U87MG cells were purchased from Elabscience (Houston, TX, USA). LN-18 and SW1088 cells were purchased from ATCC (Manassas, VA, USA). U251 cells were obtained from CLS Cell Lines Service GmbH (Eppelheim, Germany). In U87MG (p53 wild type) and in LN-18 (heterozygous for the p53 mutation Cys238Ser) p53 transcriptional activity is preserved [12, 13]. In U251 and SW1088 (both homozygous for p53 mutations) p53 transcriptional activity is lost [12, 14].

Glioblastoma primary cells were isolated from tumors histopathologically and molecularly characterized with EPIC arrays (Illumina, San Diego, CA, USA) and subsequent copy number as well as DNA methylation profiling. The procedures have been described [15, 16]. For details see also the “Supplementary Materials and Methods” file. The procedures have been approved by the Ethics Committee of the University of Napoli “Federico II” and registered as #56/21. Patient informed consent has been obtained.

SETD8 activity was inhibited using UNC0379 (Selleckchem, Houston, TX, USA) at a concentration of 5 μM, unless otherwise specified. Wee1 activity was inhibited using adavosertib (formerly, AZD1775/MK1775; Selleckchem) at a concentration of 400 nM, unless otherwise specified.

### Expression vector and siRNA transfections

Expression vector transfections were performed using Linear Polyethylenimine (L-PEI; Polysciences Inc., Warrington, PA, USA). siRNAs targeting the 3’-UTR of human SETD8 were purchased from Dharmacon Inc. (Lafayette, CO, USA). For siRNA treatment and complementation experiments LN-18 and U251 cells were transfected with non-targeting or specific siRNAs; 12 h later, cells were transfected with a pcDNA myc-Flag SETD8 plasmid (OriGene, Rockville, MD, USA).

### Gene expression database searching

SETD8 expression was explored through the Gene Expression Profiles Interactive Analysis (GEPIA) web server [17]. SETD8 expression levels, retrieved from RNA-seq data produced by the TCGA and GTeX consortia, were compared in low-grade gliomas and glioblastomas from TCGA and normal control samples from GTeX. The boxplot comparing SETD8 expression in tumor versus control samples, as well as the Kaplan-Meier curves, were also produced through GEPIA. For the comparison of SETD8 expression in glioblastomas versus low-grade glioma samples, the expression values were downloaded from the GDC data portal and analyzed in R (version 4.3).

### Real-time PCR

To assess SETD8, p21, and Chk1 transcription levels, RNA was extracted and real-time PCR was performed as described [18]. For details, see also the “Supplementary Materials and Methods” file. Primer sequences were: SETD8 Forward (FW) 5′- ACTTACGGATTTCTACCCTGTC-3′; SETD8 Reverse (REV) 5′- CGATGAGGTCAATCTTCATTCC-3′; p21 FW 5′- GCGATGGAACTTCGACTTTGT-3′; p21 REV 5′- GGGCTTCCTCTTGGAGAAGAT-3′; Chk1 FW 5′- GGTGCCTATGGAGAAGTTCAA-3′; Chk1 REV 5′- TCTACGGCACGCTTCATATC-3′. Real-time PCRs were performed in duplicate and repeated three times.

### Cell viability, FACS analysis, and caspase assay

Cell viability was investigated using the MTT test (Sigma-Aldrich), following manufacturer’s instructions. Cell viability experiments were performed in triplicate and repeated three times.

To study cell cycle distribution, cells were treated as indicated for 48 h and, then, collected, fixed with ethanol, and marked with Propidium Iodine (Merck Millipore, Burlington, MA, USA). Propidium iodine-stained cells were analyzed with the BD FACS CantoII flow cytometer and the Modfit LT 3.0 software. FACS analyses were performed in triplicate, in the case of the glioblastoma cell lines, and in duplicate, in the case of the glioblastoma primary cells.

Apoptotic cellular death was investigated using the EnzCheck Caspase-3 assay kit (Thermo Fisher Scientific), following manufacturer’s instructions. Caspase activity was inhibited by 1 mM z-VAD-FMK (included in the assay kit). Caspase assays were performed in duplicate and repeated three times.

### Dose-response plot and combination index computation

To generate the dose-response plot, cell viability was computed as the ratio of MTT signal on treated plates to the mean of MTT signal of untreated plates.

To compute the combination index, the antiproliferative effect of each drug concentration was computed as 1—cell viability [19]. The obtained values for individual drug and their combination were then analyzed through the CompuSyn 1.0 software (ComboSyn Inc., 2005).

### Immunological procedures

All the antibodies are listed in the “Supplementary Materials and Methods” file.

Immunohistochemistry was performed on a glioma tumor array (US Biomax, Inc, Rockville, MD, USA), using a specific anti-SETD8 antibody.

To perform immunofluorescence, cells were spotted on cover glass and treated with DMSO or UNC0379 or with siRNAs for 48 h. After treatment, cells were processed as described [20]. For details, see also the “Supplementary Materials and Methods” file. DNA was stained by DAPI (Sigma-Aldrich). Samples were observed and photographed using an Axiovert 200 M inverted microscope equipped with the Apotome slider module with 40× and 63× objectives (Zeiss, Oberkochen, Germany).

Immunoblot analyses of glioblastoma cells were performed upon chemical and genetic inhibition of SETD8. After 48 h of treatment, cells were collected and lysed. Lysis buffer was 0.1% NP40-PBS, plus phosSTOP and Complete Protease inhibitor (Roche Life Science, Penzberg, Germany). Cell lysates were put on ice for 10 min and centrifuged twice at 14,000 × *g* for 10 min. Supernatants were collected and protein lysates were separated on SDS/PAGE and blotted. Western blots were repeated three times. Full and uncropped western blots are uploaded in the “Supplementary Figures” file.

### Xenograft model

Five million U251 cells, engineered to stably express luciferase, were injected in the flank of 32, female, 6-week-old CD1 mice (Charles River Laboratories, Wilmington, MA, USA). The G*Power software (v. 3.1) was used to calculate the number of the sample size of each of the groups to reach statistically significant results. After 8 days of tumor implantation, 4 animals were excluded because of no sign of tumor implantation. Twenty-eight mice were then randomized into four experimental groups (each composed of 7 mice). Randomization was not blinded: the animals were divided to have animals with similar tumor sizes in each group. Animal treatments are detailed in the figure reporting the results of the xenograft experiment.

Tumor diameters were measured using a digital caliper. Tumor volume was calculated using the formula V = *A* × *B*^2^/2 (where A= major diameter, B= minor diameter).

In control mice and mice treated with UNC0379+adavosertib, bioluminescence with IVIS technology (PelkinElmer, Waltham, MA, USA) was also measured, upon luciferin injection, at the start and at the end point of the experiment. A region of interest was segmented, and the total flux (photon/s) and average radiance (photons/s/cm^2^/sr) was calculated for each animal.

The protocol was approved by the Italian Ministry of Health and registered as #188/2022-PR.

### Statistical analysis

Statistical tests were performed with Prism (Prism 7.0, GraphPad). Graphs were generated with GraphPad. Statistical significance was determined by Student’s *t*-test or one-way ANOVA, in some cases followed by post-hoc analysis. A *p* value < 0.05 was considered to indicate significance (*p < 0.05, **p < 0.01, ***p < 0.001). Error bars represent standard deviations.

## Results

### SETD8 is overexpressed in glioblastomas

**Fig. 1:**
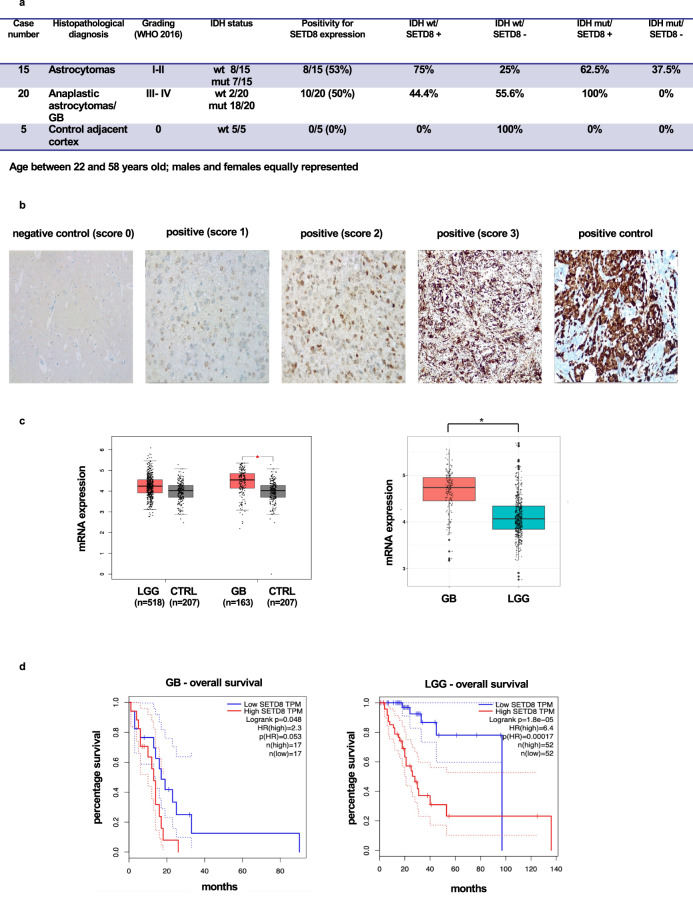
SETD8 is overexpressed in glioblastomas. **a** Table summarizing the characteristics of the samples and the results obtained from a glioma tissue microarray stained for SETD8 (abbreviations: GB: glioblastomas; wt: wild type; mut: mutated). **b** Representative images of the immunohistochemistry for SETD8 on the glioma array. From left to right: 1) normal, tumor-adjacent cortex tissue; 2) example of a glioma with a positive score 1+, this score indicating an incomplete positivity of the nucleus, in at least 50% of the analyzed cells; 3) example of a glioma with a positive score 2+, this score indicating a weak and granular positivity of the nucleus, in at least 50% of the analyzed cells; 4) example of a glioma with a positive score 3+, this score indicating an intense and granular positivity of the nucleus, in at least 50% of the analyzed cells; 5) breast cancer tissue used as positive control. **c** On the right, boxplot of SETD8 expression levels in low-grade gliomas (LGG) versus normal brain tissues (CTRL) and in glioblastoma (GB) versus normal brain tissues (CTRL). mRNA levels are reported as log2(TPM + 1). On the left, boxplot of SETD8 expression levels in GB and LGG samples. mRNA levels are reported as log2(TPM + 1). Differences with absolute log FC > 0.5 and ANOVA test *p*-value < 0.01 were considered as statistically significant. **d** On the right, Kaplan-Meier curve of overall survival (months) in glioblastoma (GB) samples with high (upper decile, red) and low (lower decile, blue) SETD8 expression. On the left, Kaplan-Meier curve of overall survival (months) in low-grade gliomas (LGG) samples with high (upper decile, red) and low (lower decile, blue) SETD8 expression.

SETD8 is overexpressed in various tumor types and its overexpression is associated with greater tumor aggressiveness and poor prognosis [4]. To investigate SETD8 expression in glioblastomas, we analyzed by immunohistochemistry SETD8 protein levels on an array of glioma tumors. As detailed in Fig. 1a, the tissue microarray contained control, tumor-adjacent, normal cortex samples, and both low- and high-grade gliomas, IDH wild type or mutated. All the normal cortex samples on the array stained negative for SETD8 expression. Noticeably, instead, we found that SETD8 was expressed in 53% of the low-grade gliomas and in 50% of the high-grade gliomas. We scored SETD8 staining from 1 (the weakest) to 3 (the most intense). In Fig. 1b, glioma samples representative of the three different scores are shown, along with a normal cortex sample, negative for SETD8 expression.

To further confirm, we analyzed SETD8 RNA expression by searching public databases and found, as shown in Fig. 1c, SETD8 RNA expression to be slightly, not significantly, higher in low-grade gliomas than in control, normal brain tissue. On the other hand, SETD8 expression was significantly higher only in glioblastomas when compared to both controls, normal brain tissues and low-grade gliomas. These results are in accordance with what previously reported in a study investigating 30 samples of gliomas where SETD8 mRNA levels were found significantly higher in tumor tissues than in adjacent normal tissues and, also, significantly higher in high-grade than in low-grade gliomas [21].

Finally, as shown in Fig. 1d, the analysis of overall survival data stored in public databases indicated that SETD8 high levels of expression correlated with a worst prognosis in low-grade gliomas and in glioblastomas.

Thus, we conclude that SETD8 expression is significantly higher only in glioblastomas when compared to both control, normal brain tissues and low-grade gliomas. Moreover, SETD8 high levels of expression correlate with a worst prognosis.

### SETD8 inhibition results in DNA damage-induced glioblastoma cell line growth arrest, regardless of p53 function

To establish whether the overexpressed SETD8 protein has a functional role in glioblastoma cells, we inhibited SETD8 methyltransferase activity in two different glioblastoma cell lines, using a specific chemical inhibitor, UNC0379 [22]. Because in neuroblastoma and in myeloma p53 is a key SETD8 substrate [10, 11], to contextually address if p53 has a role in mediating SETD8 activity also in glioblastoma, we choose LN-18 cells, in which p53 transcriptional activity is preserved, and U251 cells, in which p53 transcriptional activity is lost [12, 13].

**Fig. 2:**
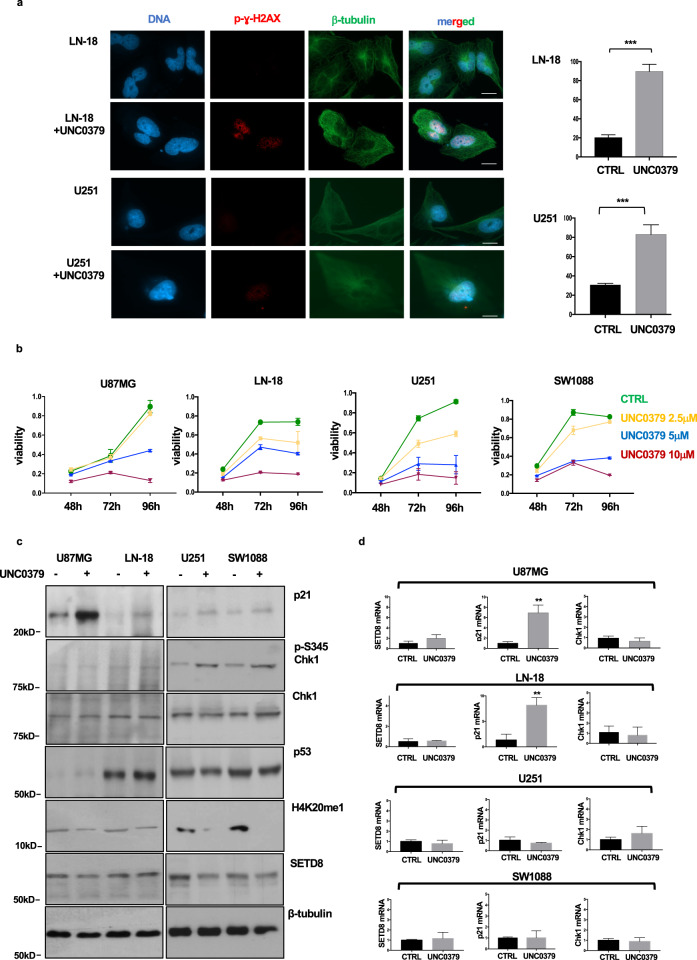
SETD8 chemical inhibition induces DNA damage and cell cycle arrest in glioblastoma cell lines. **a** LN-18 and U251 glioblastoma cell lines were treated with vehicle (DMSO) or with 5 μM UNC0379. After 48 h, cells were fixed and immunostained for p-γ-H2AX (red) to assess DNA damage and for β-tubulin (green) to label the cytoskeleton and ensure cellular integrity. DNA was stained by DAPI (blue). Representative fluorescent microscopy images are shown (scale bars: 20 μm). Graphs show percentage of cells, treated with vehicle (CTRL) or with 5 μM UNC0379, positive for p-γ-H2AX foci (cells were called positive if with ≥5 foci). Statistical analyses were performed using Student’s *t* test. **b** U87MG, LN-18, U251, and SW1088 cells were treated with DMSO (CTRL) or with the indicated UNC0379 concentrations. Cell viability was monitored by MTT assay at indicated time points. **c** Glioblastoma cell lines were treated with vehicle or with 5 μM UNC0379. After 48 h, control and UNC0379-treated cells were lysed and the extracted proteins were separated on SDS-PAGE, blotted, and probed for the indicated antigens. **d** RNA expression of SETD8, p21 and Chk1 was assessed by real-time PCR. Statistical analyses were performed using Student’s *t* test. All values are given as mean ± standard deviation of at least three replicates. ***p < 0.001; **p < 0.01.

Glioblastoma cells have high basal levels of DNA damage mostly as a consequence of replication stress [23]. As shown in Fig. 2a, as expected seen the key role of SETD8 in orchestrating DNA damage response [7], SETD8 inhibition resulted in an even greater increase of DNA damage in all the treated glioblastoma cell lines, as demonstrated by the significant high number of p-γ-H2AX foci in both proficient- and deficient-p53 cells. To investigate the functional consequences of UNC0379 treatment, we performed viability MTT assays on LN-18 and U251 cells, as well as on p53-wild type U87MG and p53-deficient SW1088 glioblastoma cells. As shown in Fig. 2b, the UNC0379-induced DNA damage resulted in a dose- and time-dependent delay of proliferative rate, as demonstrated by the viability MTT assay. Of note, higher doses of UNC0379 induced proliferation block in all the treated cells, regardless of the p53 transcriptional proficiency. More in detail, as shown in Fig. 2c, we observed, although with notable quantitative differences, p53 protein stabilization in all the analyzed cell lines, as expected seen the high basal levels of DNA damage in glioblastoma cells. However, in p53-proficient U87MG and LN-18 cell lines, SETD8 inhibition and DNA damage induced p21 accumulation and G1/S arrest. On the other hand, in U251 and SW1088 cells, which have lost p53 transcriptional activity, DNA damage induced by SETD8 inhibition resulted in G2/M arrest mediated by Chk1 phosphorylation and activation (see also the FACS profiles shown below, in the next figure and in Supplementary Fig. 1). UNC0379 treatment abrogated histone H4 methylation on lysine 20, without significantly affecting SETD8 protein levels, proving that inhibition of SETD8 activity was indeed effective (Fig. 2c).

As further proof, we analyzed SETD8, p21, and Chk1 mRNA levels in p53 proficient and deficient glioblastoma cell lines, treated for 48 h with UNC0379. As shown in Fig. 2d, in accordance with protein levels, SETD8 mRNA levels were not modified upon UNC0379 treatment. Also confirming western blot results, UNC0379 caused a significant increase in p21 transcription in p53-proficient, U87MG, and LN-18 cells but not in p53-deficient, U251, and SW1088 cells. Chk1 transcription levels were unaffected by UNC0379, further proving that SETD8 inhibition regulated Chk1 activity post-transcriptionally, inducing its phosphorylation.

**Fig. 3:**
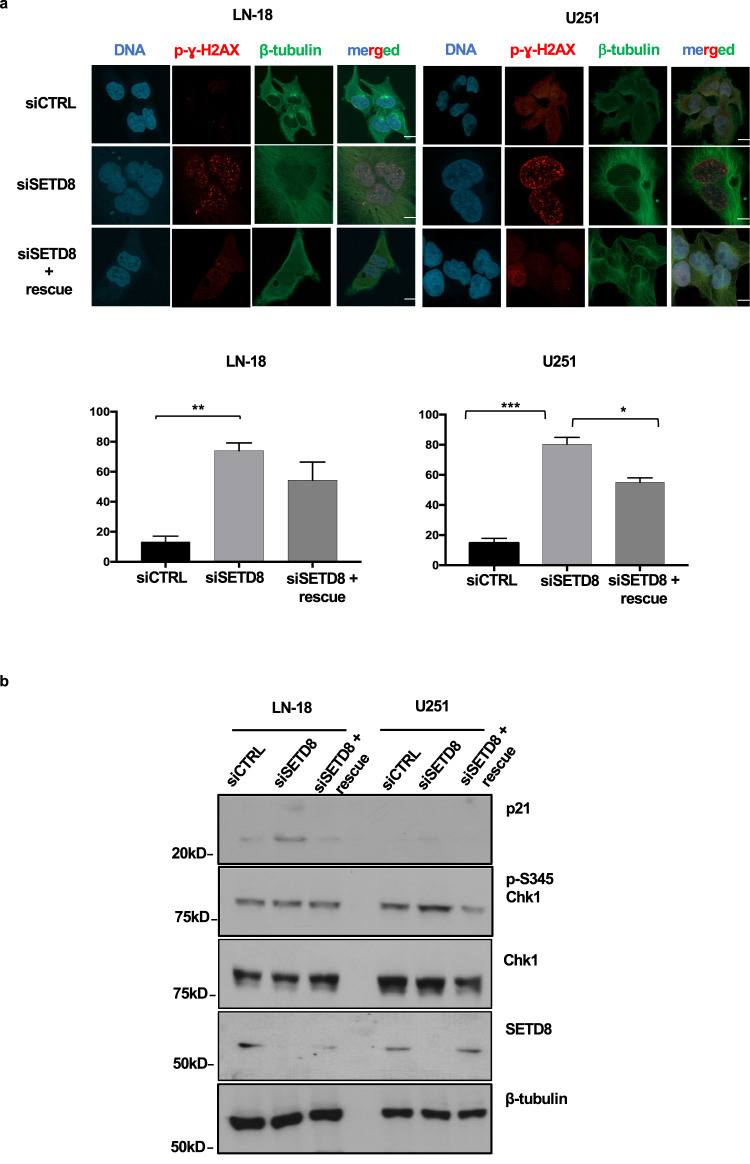
SETD8 inhibition by siRNAs induces DNA damage and cell cycle arrest in glioblastoma cell lines. **a** LN-18 and U251 cells were transfected with non-targeting siRNAs (siCTRL), with an siRNA pool targeting the SETD8 3′-UTR (siSETD8), alone or with also a plasmid expressing an siRNA resistant SETD8, pcDNA myc-Flag SETD8 (siSETD8+rescue). Cells were fixed and immunostained for p-γ-H2AX (red) to assess DNA damage and for β-tubulin (green) to label the cytoskeleton and ensure cellular integrity. DNA was stained by DAPI (blue). Representative fluorescent microscopy images are shown (scale bars: 20 μm). Graphs show percentage of cells positive for p-γ-H2AX foci (cells were called positive if with ≥5 foci). Statistical analyses were performed using Student’s *t* test. **b** A fraction of siCTRL, siSETD8 and siSETD8+rescue cells were collected and lysed. Extracted proteins were separated on SDS-PAGE, blotted and probed for the indicated antigens. All values are given as mean ± standard deviation of at least three replicates. *** *p* < 0.001; ** *p* < 0.01; * *p* < 0.05.

To further control whether the UNC0379 effects were induced by SETD8-specific inhibition, we silenced SETD8 by siRNAs in p53-proficient LN-18 cells and in p53-deficient U251 cells. As shown in Fig. 3a, SETD8 downregulation mimicked the effects of UNC0379, increasing DNA damage, as demonstrated by the significant high number of p-γ-H2AX foci, in glioblastoma cell lines, regardless of p53 status. Moreover, as shown in Fig. 3b, SETD8 silencing resulted in p21 induction in p53-proficient LN-18 cells, and in Chk1 phosphorylation in p53-deficient U251 cells. Importantly, SETD8 silencing-induced effects were rescued by transfection of a plasmid expressing the cDNA of SETD8, thus resistant to the siRNAs, directed against the 3′ untranslated region of the gene. To summarize, SETD8 silencing exactly mimicked UNC0379 effects, demonstrating the specificity of the drug.

Altogether, our results demonstrate that, in glioblastoma cells, chemical or genetic inhibition of SETD8 results in DNA damage, in turn inducing cell cycle arrest at the safeguard checkpoints. The cells arrest at the G1/S or at the G2/M checkpoint depending on p53 proficiency: p53-proficient cells arrest at the G1/S; p53-deficient cells arrest at the G2/M checkpoint.

### The SETD8 inhibitor UNC0379, in synergy with the Wee1 inhibitor adavosertib, induces death of glioblastoma cell lines

**Fig. 4:**
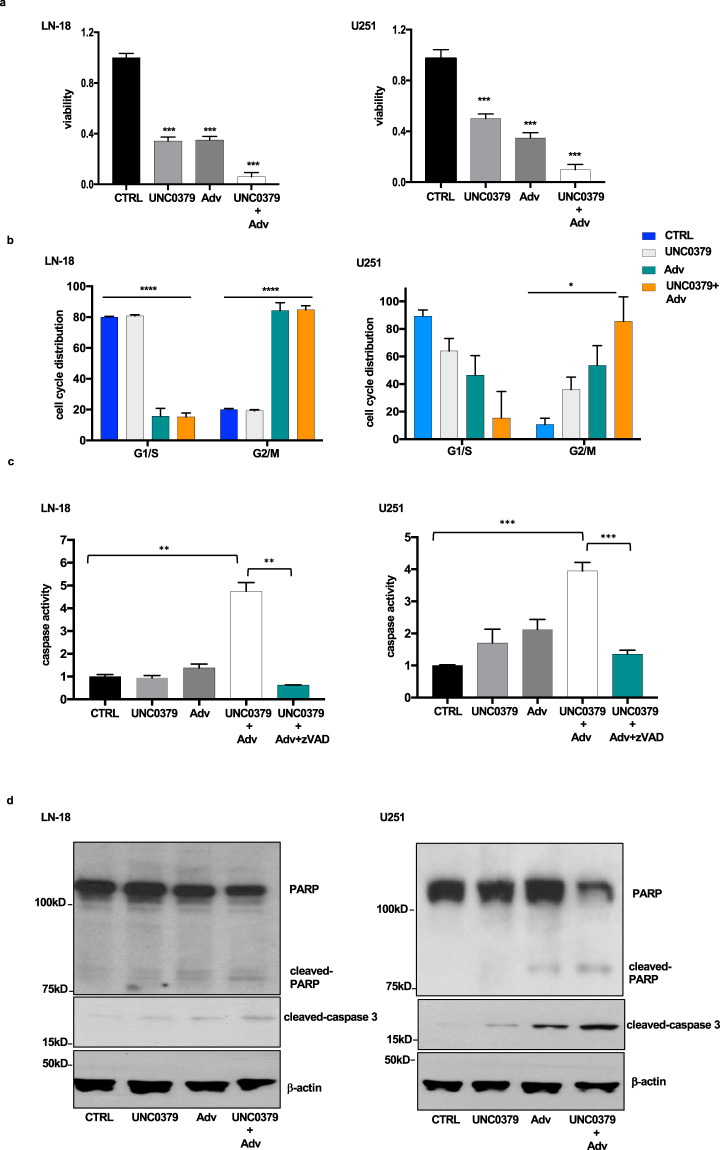
The SETD8 inhibitor UNC0379, in synergy with the Wee1 inhibitor adavosertib, induces cell death through mitotic catastrophe in glioblastoma cell lines. **a** LN-18 and U251 cells were treated with vehicle (CTRL), 5 μM UNC0379 and 400 nM adavosertib (Adv), alone or in combination, for 48 h. Cell viability was monitored by MTT assay. Statistical analyses were performed by one-way ANOVA, followed by multiple *t* test and *p* value was referred to CTRL. All values are given as mean ± standard deviation of at least three replicates. ***p < 0.001. **b** Cell cycle distribution of cells, treated as in (**a**), was monitored by FACS analysis. Percentage of cells in G1/S and G2/M is indicated. Statistical analyses were performed using one-way ANOVA, followed by multiple *t-*test. In panel, we indicated one-way ANOVA *p* value. *p* value was referred to CTRL G1/S and G2/M. ***p < 0.001; *p < 0.05. **c** Death of cells, treated as in (**a**), was assessed detecting caspase activity. To inhibit caspase activity, cells were also incubated with z-VAD-FMK (+ z-VAD) from the time of UNC0379+adavosertib addition. Statistical analyses were performed by one-way ANOVA, followed by multiple *t* test and *p* value was referred to CTRL. All values are given as mean ± standard deviation of at least three replicates. *** *p* < 0.001; ** *p* < 0.001. **d** Cells, treated as in (**a**), were lysed and death was assessed monitoring cleavage of PARP and caspase 3 proteins by western blot. Protein levels were normalized by β-actin probing.

Taking cues from our and others’ previous studies [24, 25], we reasoned that, by inhibiting the cell cycle checkpoint kinase Wee1, UNC0379-DNA damaged, cell cycle arrested cells should bypass safeguard checkpoints, progressing to mitosis, where they are expected to die by a caspase-mediated, tumor suppressor mechanism known as mitotic catastrophe [26–29]. Thus, we treated LN-18 and U251 glioblastoma cell lines with UNC0379 and adavosertib and found, as shown in Fig. 4a, that the combination of the two drugs was much more effective than each single drug in reducing viability. Importantly, FACS analyses indicated a progressive accumulation of cells in mitosis, when adavosertib was added to the SETD8 inhibitor (Fig. 4b and supplementary fig. 1). Time-lapse microscopy confirmed that LN-18 cells treated with UNC0379+adavosertib arrested in mitosis, showing morphological features of apoptosis, such as membrane blebbing and chromatin condensation (Supplementary Fig. 2).

Finally, we proved, by measuring caspase activity, that the UNC0379+adavosertib-induced decrease in cell viability was consequence of caspase-mediated cell death (Fig. 4c). As further proof, as shown in Fig. 4d, UN0379+adavosertib treatment was more effective than each single drug in inducing cleavage of PARP and caspase 3 in both LN-18 and U251 cell lines.

To investigate whether the effects of UNC0379 + adavosertib combination were synergic and not merely additive, we computed the combination index (CI) of the two drugs. Specifically, we computed the CI at a constant concentration of 5 μM of UNC0379 and scalar concentrations of adavosertib, ranging between 0.1 μM and 1.2 μM. As shown in Supplementary Fig. 3a, 5 μM UNC0379 enhanced the anti-viability effects of adavosertib at any tested concentration. Importantly, as shown in Supplementary Fig. 3b, the CI values were below 1 (mean CI: 0.57 ± 0.13) along the entire range of combined drug concentrations, thus indicating a synergistic effect of the UNC0379 and adavosertib combination.

Altogether, our results demonstrate that the SETD8 inhibitor UNC0379 synergizes with the Wee1 inhibitor adavosertib in pushing DNA-damaged glioblastoma cell lines into mitosis, where they die by the caspase-mediated, genome stability safeguarding, mitotic catastrophe mechanism.

### The SETD8 inhibitor UNC0379, in synergy with the Wee1 inhibitor adavosertib, induces death of glioblastoma primary cells

**Fig. 5:**
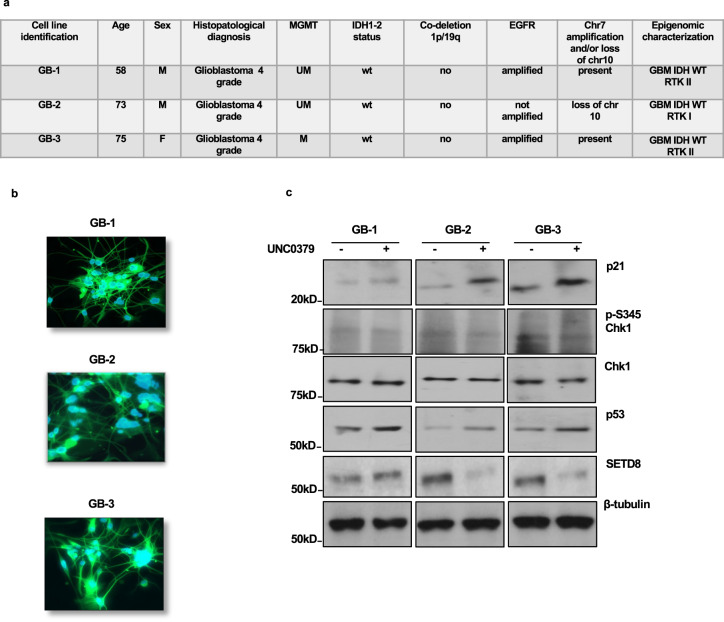
UNC0379 induces p21 protein levels in p53-proficient glioblastoma primary cells. **a** Information about the patients, the histopathological features, and the epigenetic profile of glioblastoma tissues from which three primary cell lines (GB-1, GB-2, and GB-3) have been isolated (abbreviations: chr: chromosome; M: male; F: female; UM: un-methylated; M: methylated; wt: wild type; mut: mutated). **b** Immunocharacterization with GFAP antibody of the three primary cell lines. DNA was stained by DAPI. **c** Glioblastoma primary cells were treated with vehicle or with 5 μM UNC0379. After 48 h, control and UNC0379-treated cells were lysed and the extracted proteins were separated on SDS-PAGE, blotted and probed for the indicated antigens.

The UNC0379+ adavosertib synergistic effect was confirmed in three primary glioblastoma cell lines, isolated from tumors fully characterized, as shown in Fig. 5a, by histopathological features and methylome analysis, and expressing, as shown in Fig. 5b, the specific glial marker GFAP. The three primary glioblastoma cell lines were negative for mutations in the p53 DNA binding domain (data not shown). Accordingly, as shown in Fig. 5c, UNC0379 treatment resulted in p53 stabilization and p21 induction and but not in Chk1 activation.

**Fig. 6:**
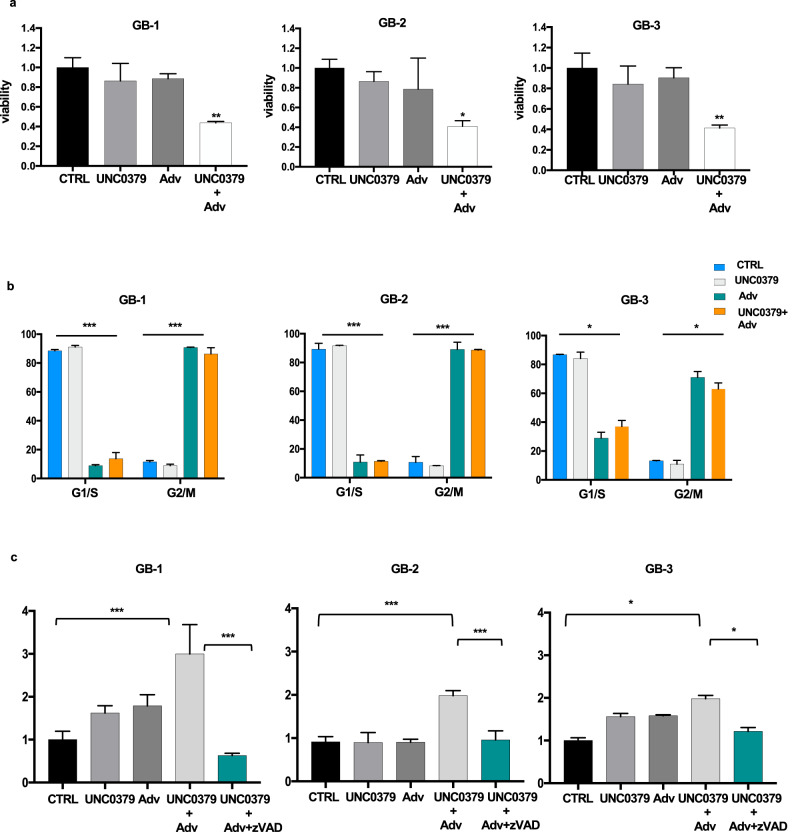
UNC0379+adavosertib combination induces cell death through mitotic catastrophe in glioblastoma primary cells. **a** Each primary cell line (GB-1, GB-2, and GB-3) was treated with 5 μM UNC0379 and 400 nM adavosertib, alone or in combination, for 48 h and compared with vehicle-treated control cells (CTRL). Cells viability was assessed using the MTT assay. Statistical analyses were performed using one-way ANOVA, followed by multiple comparison. *p* value was referred to CTRL. **p < 0.01; *p < 0.05. **b** Cell cycle distribution of cells, treated as in (**a**), was monitored by FACS analysis. Percentage of cells in G1/S and G2/M is indicated. All values are given as mean ± standard deviation of at least three replicates. Statistical analyses were performed using one-way ANOVA, followed by multiple comparison. In panel we indicated the one-way ANOVA *p* value. *p* value was referred to G1/S and G2/M in CTRL cells. ***p < 0.001; *p < 0.05. **c** Each primary cell line was treated with 5 μM UNC0379 and 400 nM adavosertib, alone or in combination, for 72 h, and compared with vehicle-treated control cells. Death of cells was monitored by detecting caspase activity. To inhibit caspase activity, cells were also incubated with z-VAD-FMK (+ z-VAD) from the time of UNC0379+adavosertib addition. All values are given as mean ± standard deviation of at least three replicates. Statistical analyses were performed using one-way ANOVA, followed by multiple comparison. *p* value was referred to CTRL. ***p < 0.001; *p < 0.05.

As shown in Fig. 6a, also in glioblastoma primary cells, in agreement with results obtained in cell lines, UNC0379+adavosertib treatment significantly reduced viability. FACS analyses indicated a progressive accumulation of all the three investigated primary cells in mitosis, when adavosertib was added to the SETD8 inhibitor (Fig. 6b and supplementary fig. 4). As shown in Fig. 6c, UNC0379 and adavosertib combination resulted also in substantial potentiation of caspase activation and apoptotic cell death compared with single treatments.

Altogether, our results demonstrate that the SETD8 inhibitor UNC0379 synergizes with the Wee1 inhibitor adavosertib in inducing caspase-mediated cell death in mitosis also of primary glioblastoma cells.

### UNC0379+adavosertib treatment restrains glioblastoma growth in a xenograft mouse model

**Fig. 7:**
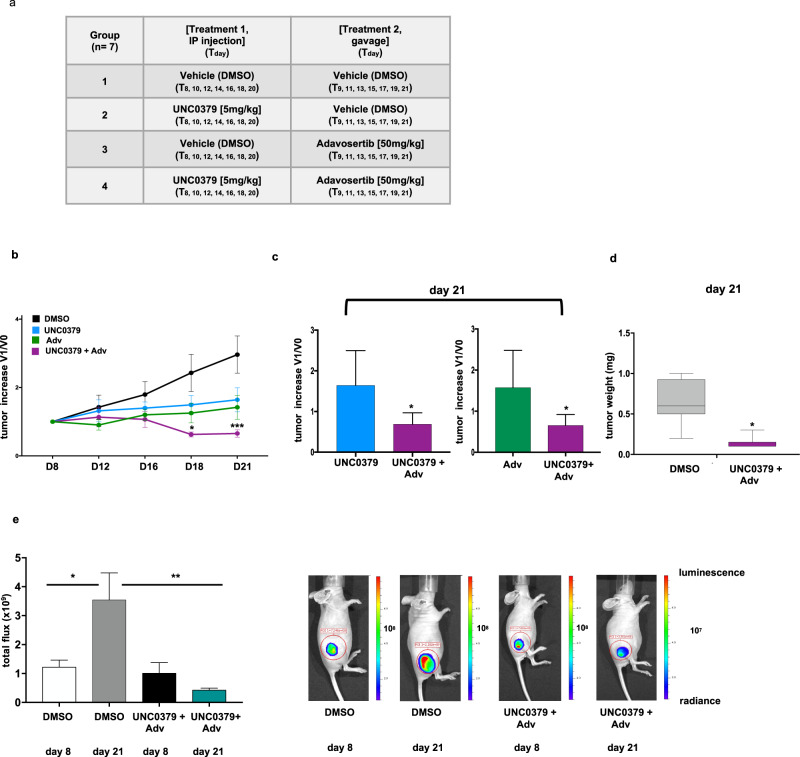
The SETD8 inhibitor UNC0379, in combination with the Wee1 inhibitor adavosertib, impairs glioblastoma tumor growth in a xenograft mouse model. **a** U251 glioblastoma cells were inoculated in CD1 mice. After 8 days, mice were randomized into four experimental groups and treated as indicated. **b** Tumor dimensions were measured using a digital caliper. Dimensions (V1) at each time point (D8–D21) were compared to dimensions at treatment start point (V0) to report tumor increase. All values are given as mean ± standard error among the seven animals in each group. Statistical analysis was performed using one-way ANOVA, followed by multiple *t* test. *p* value was referred to the DMSO-treated group. **c** At day 21 (end point of the experiment) tumor increase in mice treated with UNC0379 or adavosertib alone was compared to tumor increase in mice treated with UNC0379+adavosertib in combination. All values are given as mean ± standard deviation. Statistical analysis was performed using Student’s *t* test. **d** Tumor weight, upon excision at day 21, of control or UNC0379+adavosertib-treated mice. All values are given as mean ± standard deviation. Statistical analysis was performed using Student’s *t* test. **e** Tumor growth measured, upon luciferin injection, by IVIS in vivo imaging at the start (day 8) and at end (day 21) points of the treatment. Graphs show, at both time points, total flux of control, vehicle-treated mice compared to total flux of mice treated with UNC0379+adavosertib in combination. All values are given as mean ± standard deviation. Statistical analysis was performed using Student’s *t* test. On the left, representative images of one control and of one UNC0379+adavosertib-treated mouse. *** *p* < 0.001; ** *p* < 0.01; * *p* < 0.05.

To further validate the promising, so far observed effects, we tested UNC0379+adavosertib combination in a glioblastoma xenograft animal model. We injected U251 glioblastoma cells, that better recapitulate the histology of glioblastomas when inoculated subcutaneously [30], in the flank of CD1 nude mice. Animals were treated as illustrated in Fig. 7a. As shown in Fig. 7b, both UNC0379 and adavosertib reduced glioblastoma growth but, remarkably, the combination of UNC0379+adavosertib virtually abrogated growth. As shown in Fig. 7c, the UNC0379+adavosertib combination was significatively more effective than each single drug in reducing tumor growth. To further proof, at the end point of the experiment, tumors excised from UNC0379+adavosertib-treated mice were weighted and compared to tumors excised from control mice, confirming, as shown in Fig. 7d, the statistically significant efficacy of the treatment in reducing growth.

The reliability of the above-described measurements, made by digital caliper, was controlled and confirmed, as shown in Fig. 7e, by bioluminescent optical imaging, taking advantage of the fact that U251 cells were stably transfected with a vector expressing the luciferase gene.

Thus, our data strongly prove that UNC0379+adavosertib combination is effective in inhibiting growth in a glioblastoma animal model.

## Discussion

In this report, we have shown that the lysine methyltransferase SETD8 is overexpressed in glioblastoma and is a candidate target for its treatment. As found in other tumor types [10, 11], the inhibition of SETD8 enzymatic activity results in DNA damage and cell cycle arrest at safeguard cell cycle checkpoints. Of note, differently from neuroblastoma but in accordance to what was observed in myeloma [10, 11], we have here demonstrated that DNA damage, induced by SETD8 inhibition, arrests glioblastoma cell cycle progression regardless of p53 proficiency: p53-proficient cells arrest at G1/S; p53-deficient cells arrest at G2/M checkpoint.

**Fig. 8:**
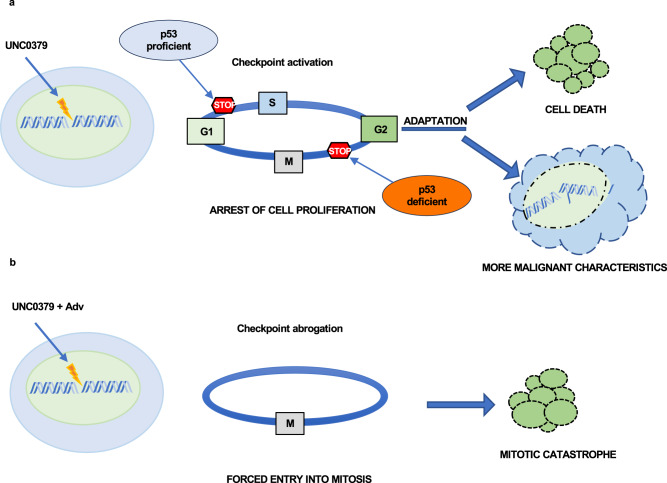
Schematic diagram depicting the effect of UNC0379, alone or in combination with adavosertib, on glioblastoma. **a** UNC0379 treatment, by inhibiting DNA repair, results in DNA damage accumulation in glioblastoma cells. DNA-damaged p53 proficient cells arrest at the G1/S cell cycle checkpoint; DNA-damaged p53 deficient cells arrest at the G2/M checkpoint; however, some cells can adapt to the checkpoints and restart proliferation. Most of the cells are expected to die in subsequent cell cycles because of the high levels of DNA damage but some cells can survive acquiring more malignant characteristics. **b** Adavosertib abrogates the cell cycle checkpoints, pushing the cells with UNC0379-induced DNA damage into mitosis where they die by mitotic catastrophe, without the chance of adaptation and survival.

Proliferation arrest at cell cycle checkpoints might not, however, be a lasting successful strategy to target cancer (Fig. 8). After a prolonged arrest, cells adapt to the cell cycle checkpoints, regaining proliferation despite the presence of DNA damage [31]. Cells adapt not only to the G1/S or G2/M checkpoints but also to the mitotic spindle assembly checkpoint, a phenomenon known as mitotic slippage [32]. Although most of the checkpoint-adapted cells are expected to die in subsequent cell cycles because of excessive DNA damage, some of them might survive and continue to proliferate. Thus, DNA-damaged, checkpoint-adapted cells appear to die in a slow, stochastic way [31, 33]. Even more dangerous, checkpoint-adapted cancer cells, that survive DNA damage, might potentially accumulate further genomic instability, ultimately resisting to therapy and acquiring a more aggressive phenotype [31, 33].

Thus, to more efficaciously target glioblastoma, we reasoned that combining the SETD8 inhibitor UNC0379 with the Wee1 inhibitor adavosertib could be a successful strategy. Adavosertib abrogates both G1/S and G2/M checkpoints, triggering untimely, premature activation Cdk2 and Cdk1, respectively [27]. Thus, we predicted that adavosertib would have rapidly pushed SETD8-inhibited, DNA-damaged cells into mitosis. In turn, cells, prematurely entered into mitosis with unrepaired DNA damage, were expected to die by mitotic catastrophe, without any chance of slow adaptation [28, 29]. As further rationale, following our previous research, we have also pondered that Wee1 inhibition should have delayed slippage, extending mitosis and, thus, increasing changes in mitotic cell death [24]. Indeed, our results do prove that adavosertib synergizes with UNC0379 in inducing caspase-mediated, mitotic death of glioblastoma cell lines and primary cells. Promisingly, the combination of UNC0379+adavosertib was highly efficient in restraining glioblastoma growth also in a xenograft animal model.

Adavosertib is currently being evaluated in clinical trials, alone or in combination [34, 35]. Summarizing the results of the few already completed trials is complex, seen the relevant divergences in tumor types and stages and in combinatorial strategies. Generally speaking, adavosertib potentiates the activity of standard chemotherapies and/or radiotherapy against various tumors. However, the toxicity of the combination of adavosertib with other regimens might be a limiting factor, especially when the additional therapeutic efficacy appears to be modest. Thus, the need to identify biomarkers that can predict better responses. Our data, demonstrating that the UNC0379+adavosertib combination is highly efficacious in both p53-proficient and -deficient glioblastoma cells, establish that p53 status cannot be utilized as a biomarker for predicting response, making, on the other hand, UNC0379+adavosertib treatment an option for the high percentage of glioblastomas with impaired p53 pathway [36]. Conversely, our studies suggest that SETD8 overexpressing glioblastomas could be more specifically targeted by the novel UNC0379+adavosertib combination. Further studies are ongoing in our laboratory to formally and definitively prove that SETD8 overexpression could, indeed, be a biomarker, easily investigated by immunohistochemistry, for predicting response of glioblastomas to UNC0379+adavosertib combination.

Non-negligible, adavosertib has already been investigated also for the treatment of tumors of the central nervous system, proving that it can penetrate across the human blood–brain barrier [37, 38]. The lipophilic structure and the molecular weight of UNC0379 also suggest permeability across the blood–brain barrier [39], a property that will require, however, further verification with ad hoc in vitro and in vivo studies.

The ectopic mouse model we used allowed a first assessment of the efficiency of the novel combinatorial UNC0379+adavosertib therapy against glioblastoma cells in vivo. It was also, importantly, instrumental to rule out acute toxicity of the treatment. However, the response to UNC0379+adavosertib has to be tested also in an orthotopic xenograft mouse model to explore if the drugs do cross blood–brain barrier in concentrations sufficient to elicit a therapeutic response.

In conclusion, based on all the above considerations, our data strongly encourage further studies of the novel UNC0379+adavosertib combinatory approach for glioblastoma treatment, firstly in orthotopic glioblastoma models: an indispensable step forward to later, eventually, translate the treatment to the clinic.

### Supplementary information

Supplementary figures

Supplementary figures

Supplementary Materials and Methods

## Data Availability

The GBM and LGG cancer datasets analyzed during the current study are available in the TCGA data portal (https://portal.gdc.cancer.gov). The GTeX control brain data analyzed during the current study are available in the GTeX data portal (http://gtexportal.org/home).
