# Supplementary material for: Targeted inhibition of the methyltransferase SETD8 synergizes with the Wee1 inhibitor adavosertib in restraining glioblastoma growth
Source: Cell Death Dis. 2023 Sep 27;14(9):638. doi: 10.1038/s41419-023-06167-3 (PMC10533811; doi:10.1038/s41419-023-06167-3)
Supplement: Supplementary file 1 — Supplementary figures [file 41419_2023_6167_MOESM1_ESM.pdf]

## Supplementary figure 1

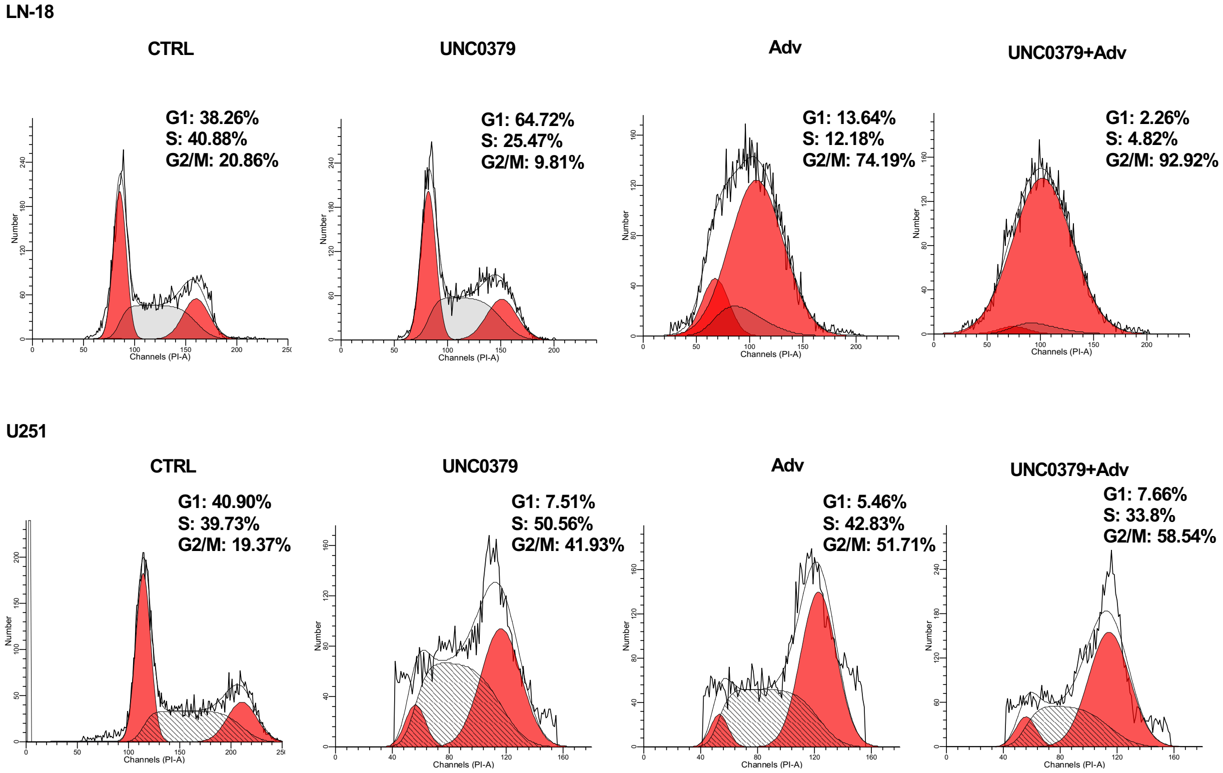


**Glioblastoma cell lines, treated with UNC0379+adavosertib, arrest in mitosis**

LN-18 and U251 cells were treated with vehicle (CTRL), 5µM UNC0379 and 400nM adavosertib (Adv), alone or in combination, for 48h. Cell cycle distribution of cells was monitored by FACS analysis. Representative cell cycle distribution graphs are shown. The percentage of cells in G1, S or G2/M phase is also indicated.

**Supplementary figure 2**

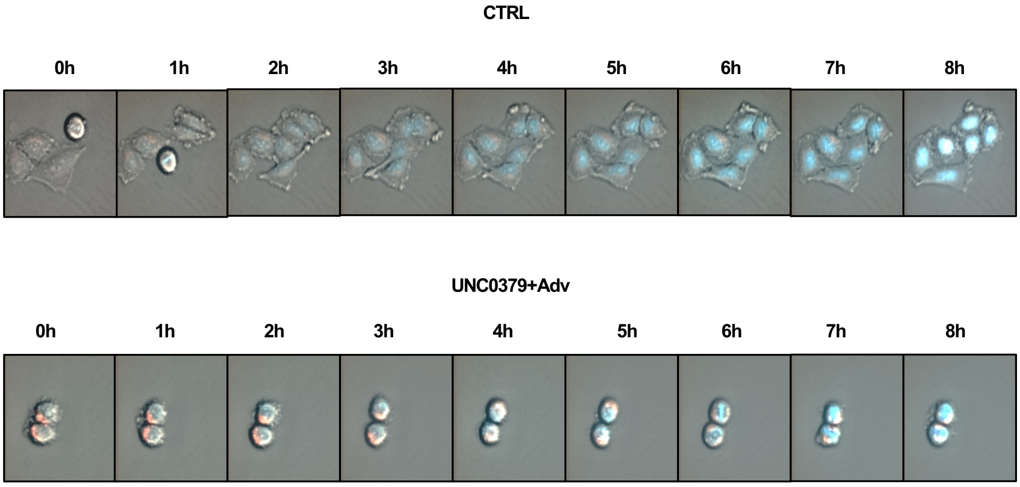


**UNC0379+adavosertib-treated glioblastoma cells arrest in mitosis and die by apoptosis**

Representative live cell images of LN-18 cells treated with DMSO+adavosertib (CTRL) or with UNC0379+adavosertib (UNC0379+Adv). Cells were monitored by time-lapse microscopy for 8 hours and pictures were taken every hour.

**Supplementary figure 3**

a

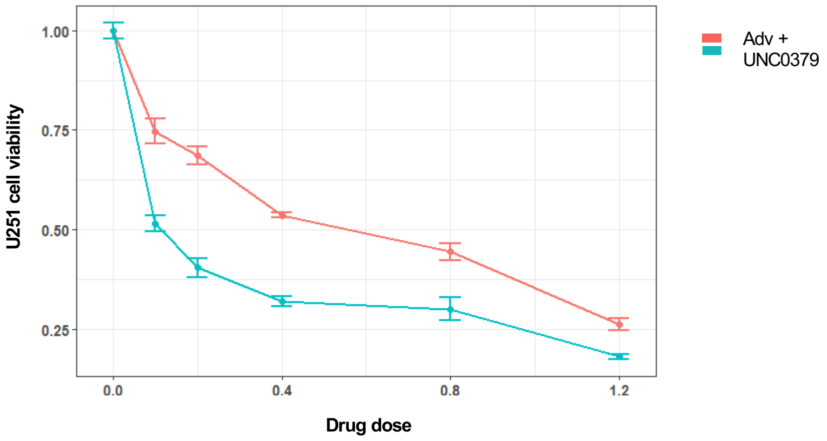


b

| Dose Adv | Dose UNC0379 | Effect | CI | Dose Adv | Dose UNC0379 |
|---|---|---|---|---|---|
| 0.1µM | 5µM | 0.48 (0.02) | 0.65 (0.07) | 0.42 (0.04) | 12.18 (1.26) |
| 0.2µM | 5µM | 0.6 (0.02) | 0.5 (0.05) | 0.74 (0.08) | 21.91 (2.49) |
| 0.4µM | 5µM | 0.68 (0.01) | 0.48 (0.04) | 1.18 (0.11) | 35.46 (3.26) |
| 0.8µM | 5µM | 0.7 (0.02) | 0.76 (0.11) | 1.9 (0.18) | 38.65 (5.78) |
| 1.2µM | 5µM | 0.82 (0.01) | 0.45 (0.03) | 3 (0.25) | 92.23 (7.99) |

**The UNC0379+adavosertib combination has a synergistic effect on glioblastoma cells**
**a** Dose-response plot of U251 cells treated with adavosertib or UNC0379+adavosertib. UNC0379 concentration was 5µM; adavosertib concentrations are indicated. Cell viability was computed as the ratio of MTT signal on treated plates to the mean of the MTT signal of untreated plates. **b** Combination index (CI) value at different combinations of adavosertib and UNC0379 is shown, along with the predicted concentrations of the two inhibitors alone needed to observe a given antiproliferative effect in U251 cells.

**Supplementary figure 4**

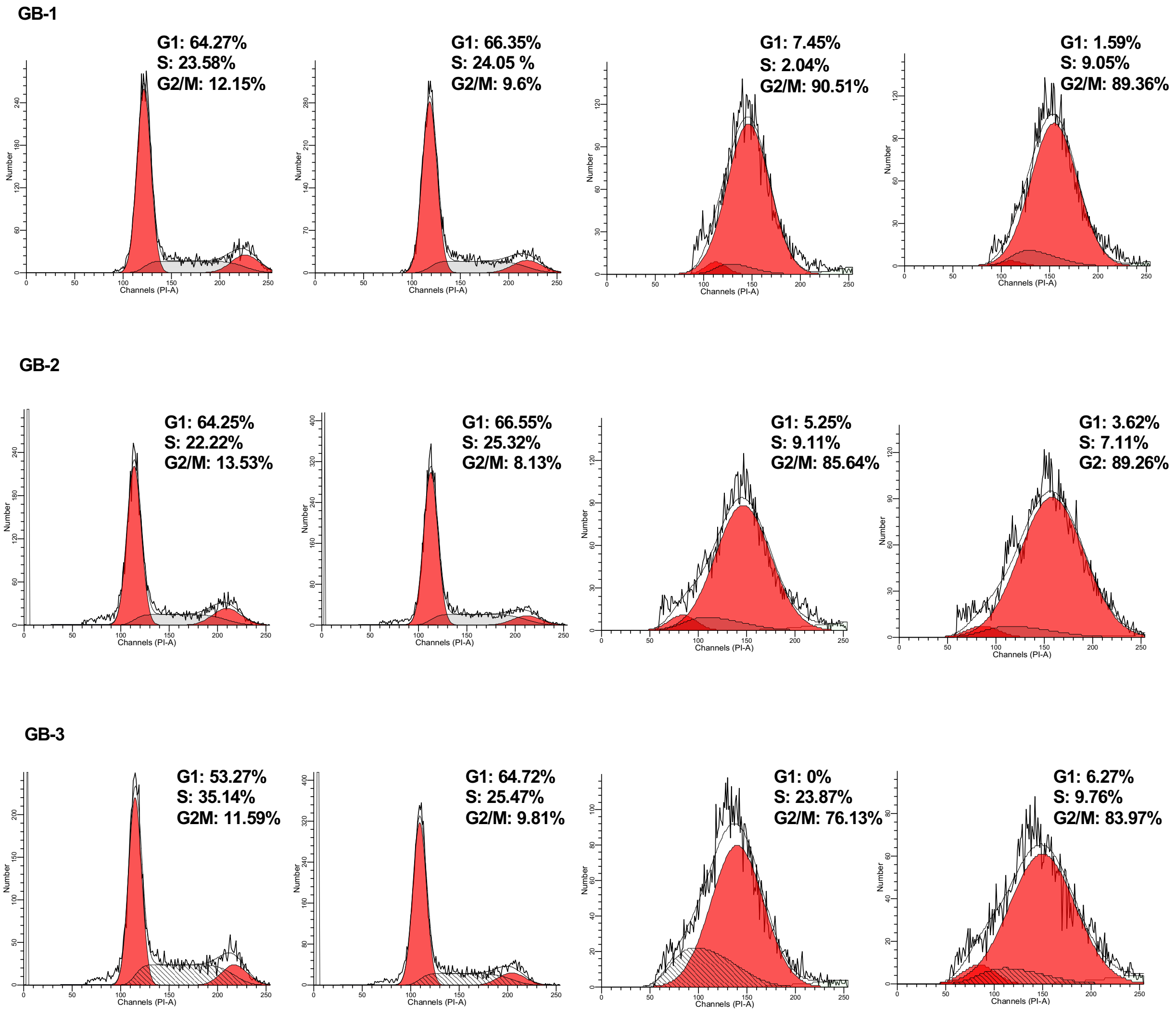


**Glioblastoma primary cells, treated with UNC0379+adavosertib, arrest in mitosis**

Three primary glioblastoma cell lines (GB-1, GB-2 and GB-3) were treated with vehicle (CTRL), 5μM UNC0379 and 400nM adavosertib (Adv), alone or in combination, for 48h. Cell-cycle distribution of cells was monitored by FACS analysis. Representative cell cycle distribution graphs are shown. The percentage of cells in G1, S or G2/M phase is also indicated.
