# Supplementary material for: Targeted inhibition of the methyltransferase SETD8 synergizes with the Wee1 inhibitor adavosertib in restraining glioblastoma growth
Source: Cell Death Dis. 2023 Sep 27;14(9):638. doi: 10.1038/s41419-023-06167-3 (PMC10533811; doi:10.1038/s41419-023-06167-3)

Full and uncropped western blot for Fig 2C

p-Chk1 U87MG and LN-18 lanes 1-2-3-4

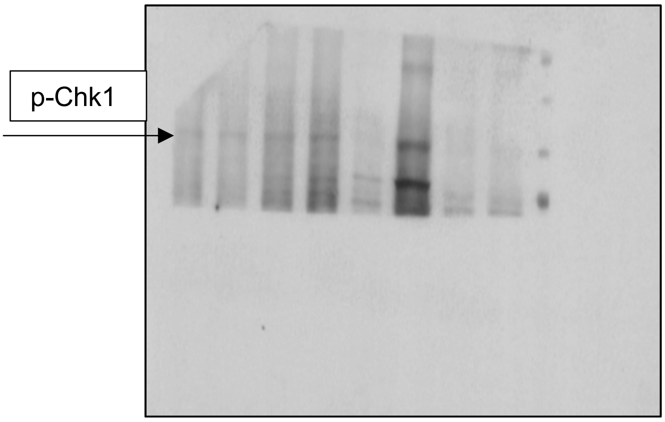

Full and uncropped western blot for Fig 2C
P-Chk1 U251 SW10-88 lanes 5-6-7-8

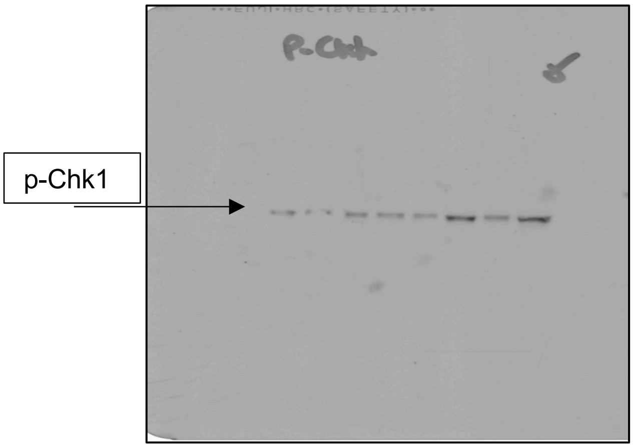

Full and uncropped western blot for Fig 2C
Chk1 U87MG Ln18 lanes 2-3-4-5

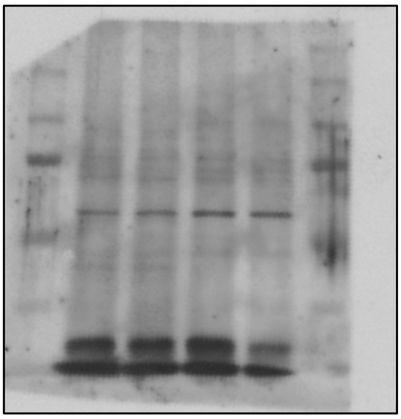

Full and uncropped western blot for Fig 2C
Chk1 U251 SW1088 lanes 5-6-7-8

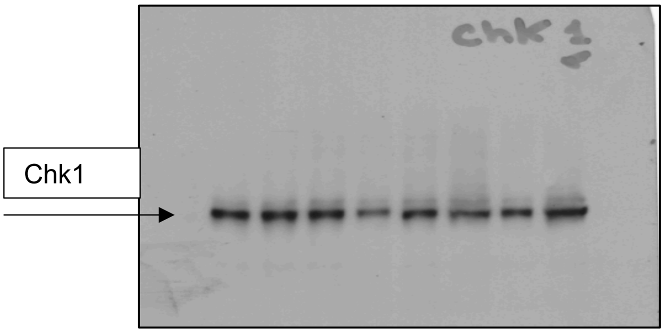

Full and uncropped western blot for Fig 2C
Tp53 U87MG and LN-18 lanes 1-2-3-4

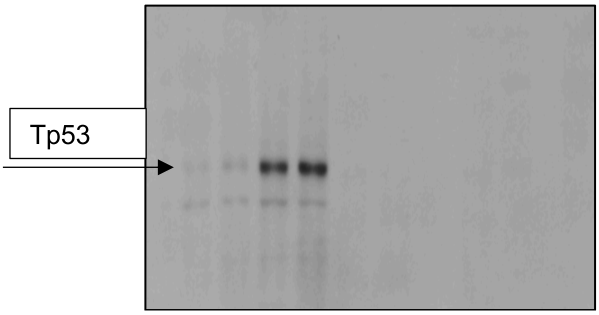

Full and uncropped western blot for Fig 2C
Tp53 U251 SW1088 lanes 5-6-7-8

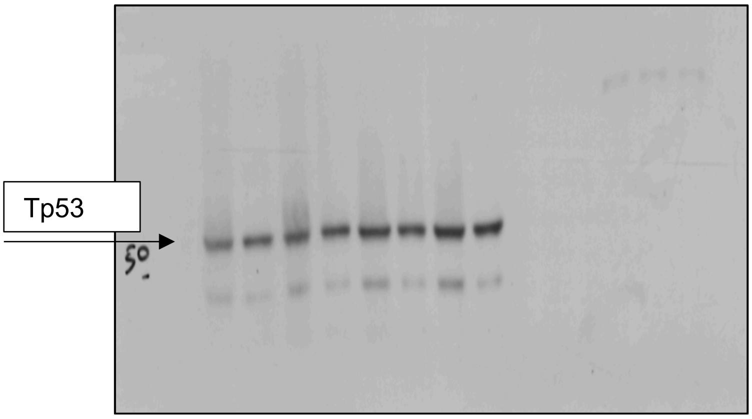

Full and uncropped western blot for Fig 2C
SETD8 U251 SW1088 lanes 1-2-3-4

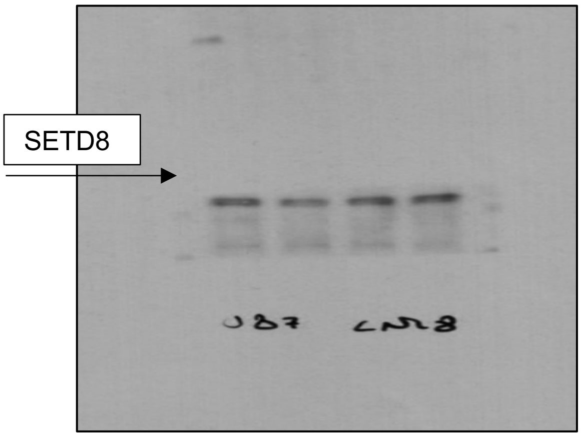

Full and uncropped western blot for Fig 2C
SETD8 U251 SW1088 lanes5-6-7-8

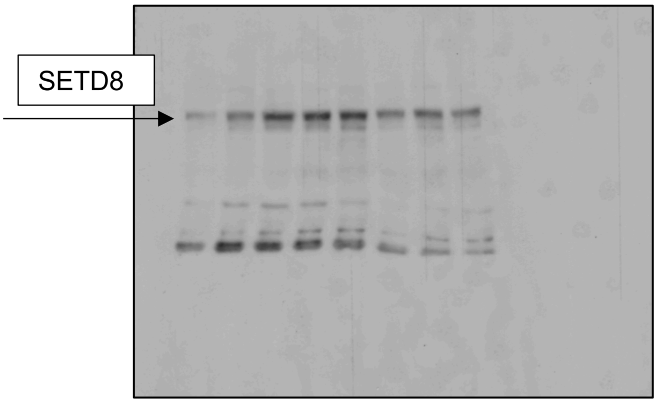

Full and uncropped western blot for Fig 2C
b-tubulin U87MG LN-18 lanes 1-2-3-4

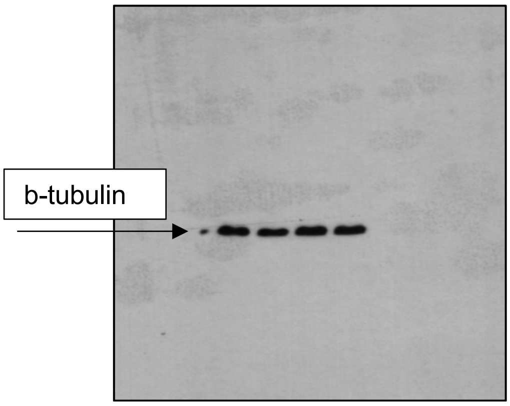

Full and uncropped western blot for Fig 2C
b-tubulin U251 SW1088 lanes 5-6-7-8

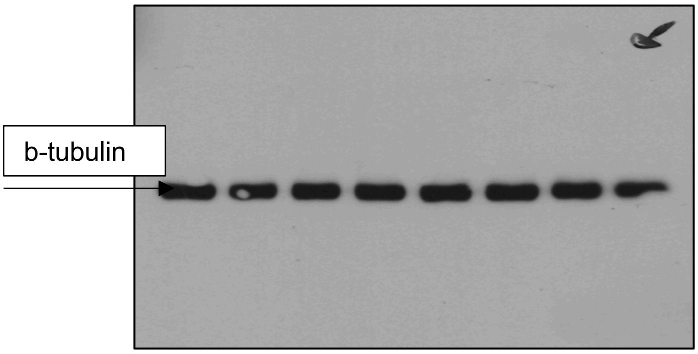

Full and uncropped western blot for Fig 2C
p21 U87 Ln18 lanes 1-2-3-4

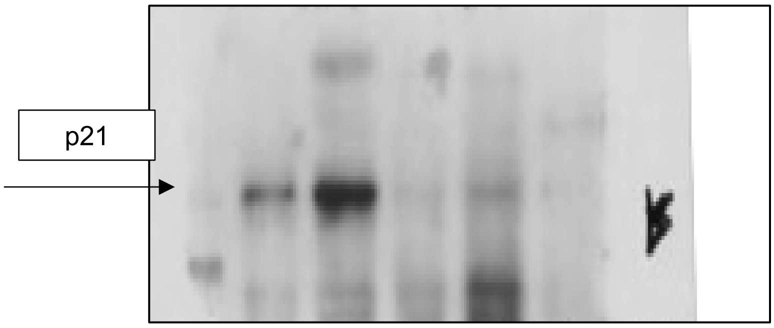

Full and uncropped western blot for Fig 2C
p21 U251 SW1088 lanes 5-6-7-8

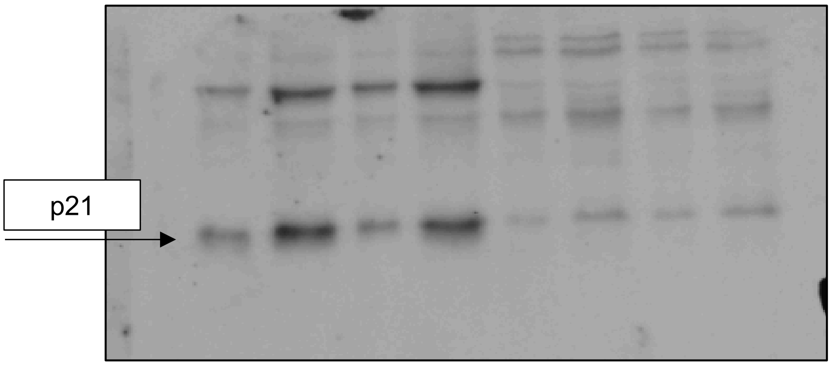

Full and uncropped western blot for Fig 2C
H4K20me1 U87MG and LN-18 lanes 1-2-3-4

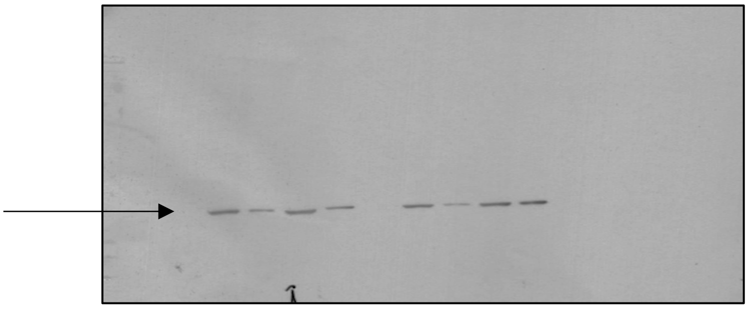

Full and uncropped western blot for Fig 2C
H4K20me1 U251 and SW1088 lanes 1-2-3-4

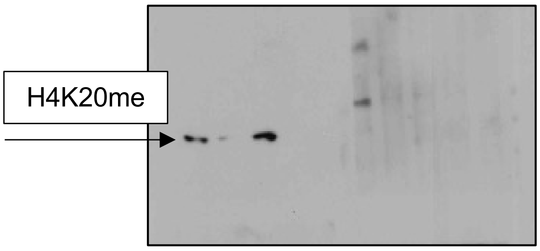

Full and uncropped western blot for Fig 3B
p-S345 Chk1 LN18 (lanes 1-2-3) - U251 (lanes 5-6-7)

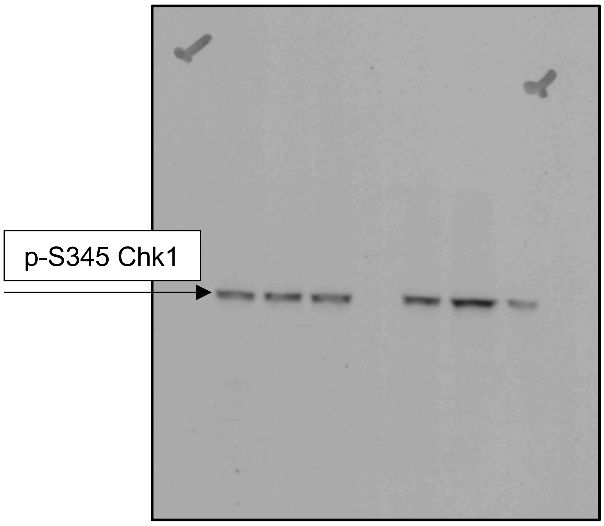

Full and uncropped western blot for Fig 3B
Chk1 LN18 (lanes 1-2-3) – U251 (lanes 5-6-7)

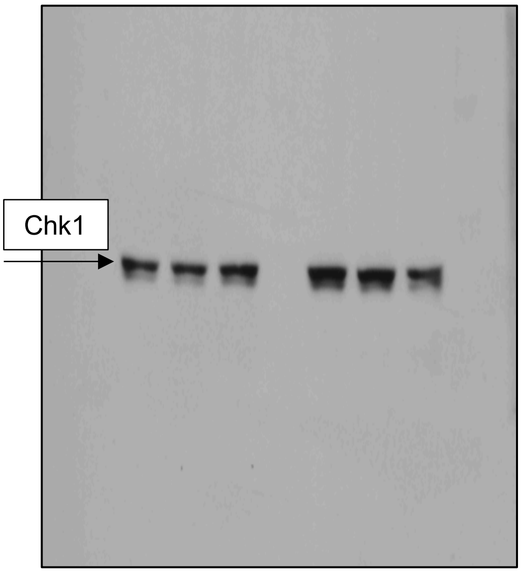

Full and uncropped western blot for Fig 3B
SETD8 LN18 (lanes 1-2-3) – U251 (lanes 5-6-7)

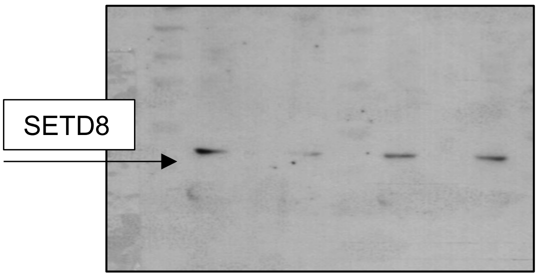

Full and uncropped western blot for Fig 3B
b-tubulin LN18 (lanes 1-2-3) – U251 (lanes 5-6-7)

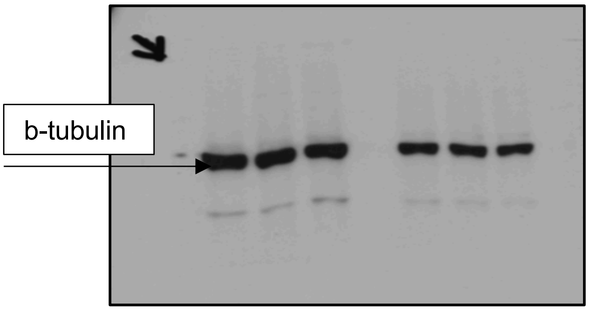

Full and uncropped western blot for Fig 3B
p21 LN18 (lanes 1-2-3) – U251 (lanes 5-6-7)

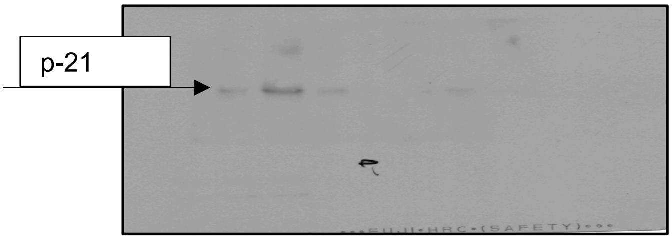

Full and uncropped western blot for Fig 4D

PARP and cleaved-PARP CTRL UNC0379 Adv UNC0379+Adv LN-18 (lanes 5-6-7-8)

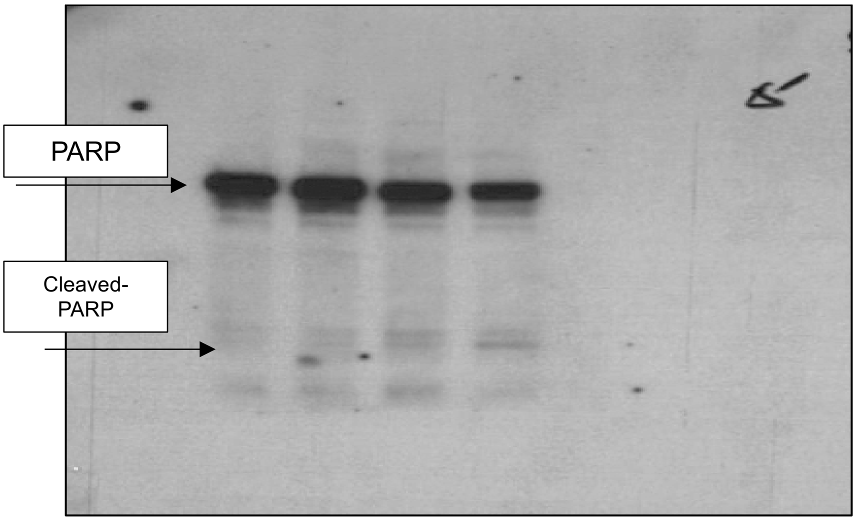

Full and uncropped western blot for Fig 4D

PARP and cleaved-PARP CTRL UNC0379 Adv UNC0379+Adv U251 (lanes 5-6-7-8)

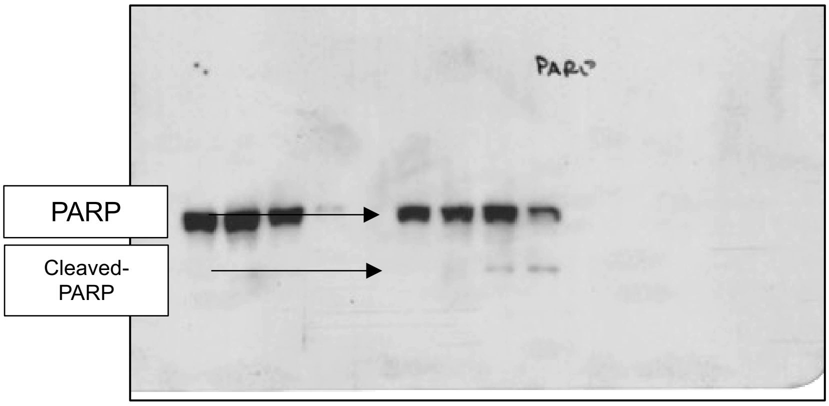

Full and uncropped western blot for Fig 4D

Actin (U251 lanes 1-2-3-4) (LN-18 lanes 5-6-7-8) CTRL UNC0379 Adv UNC0379+Adv

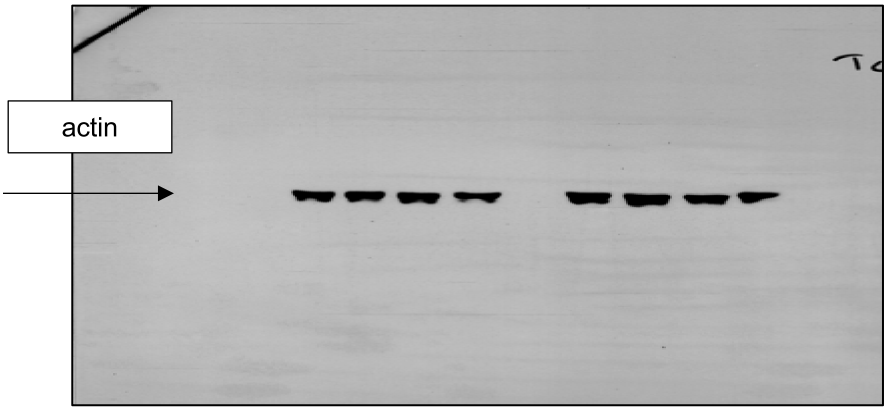

Full and uncropped western blot for Fig 4D
Cleaved-caspase 3 LN-18 (CTRL UNC0379 Adv UNC0379+Adv)

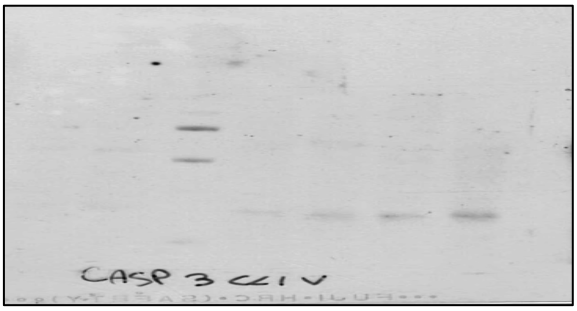

Full and uncropped western blot for Fig 4D
Cleaved-caspase 3 U251 (CTRL UNC0379 Adv UNC0379+Adv)

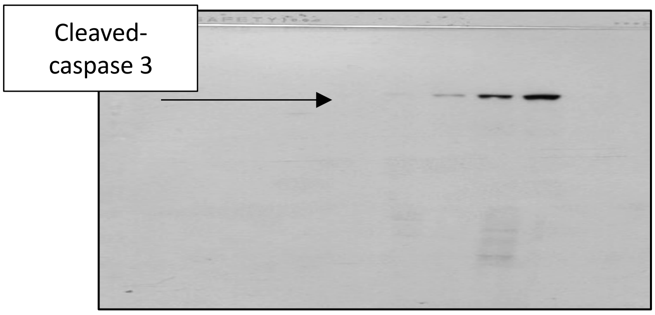

Full and uncropped western blot for Fig 5C
p-Chk1 GB-1 (lanes1-2) GB-2 (lanes 4-5) GB-2 (lanes 7-8)

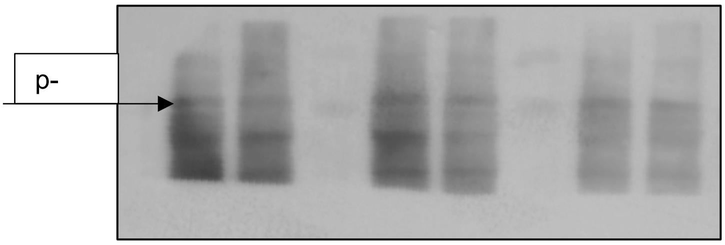

Full and uncropped western blot for Fig 5C
Chk GB-1 (lanes1-2) GB-2 (lanes 4-5)

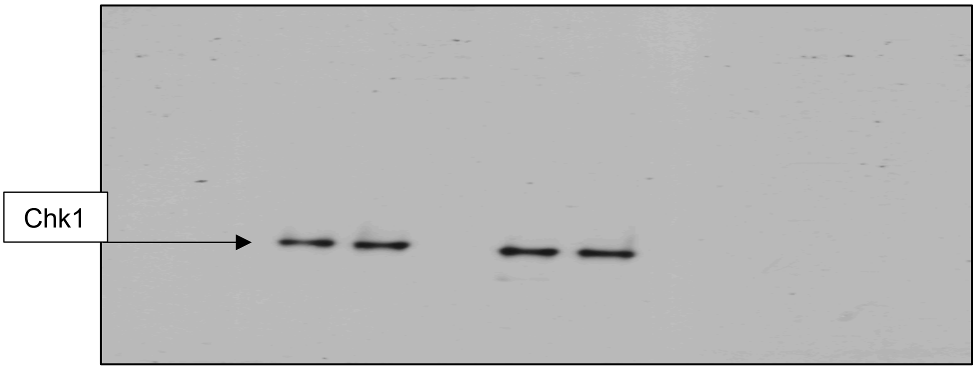

Full and uncropped western blot for Fig 5C
Chk GB-3 (lanes 1-2)

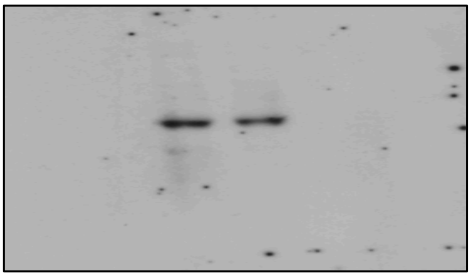

Full and uncropped western blot for Fig 5C
Tp53 GB-1 (lanes 1-2)

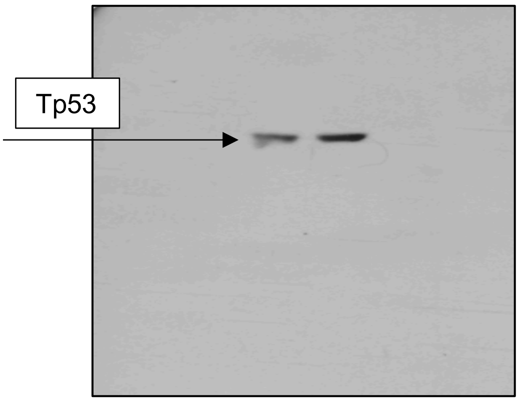

Full and uncropped western blot for Fig 5C
Tp53 GB-2 (lanes 1-2) GB-3 (4-5)

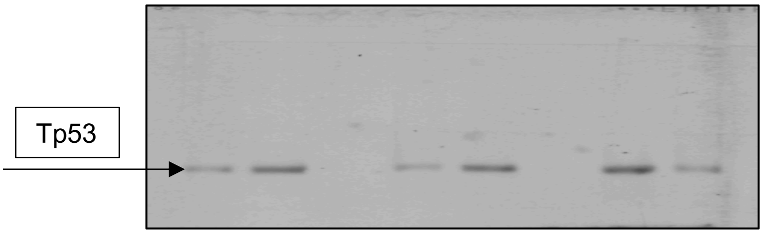

Full and uncropped western blot for Fig 5C
SETD8 GB-1 (lanes 1-2) GB-2 (lanes 4-5) GB-3 (lanes 7-8)

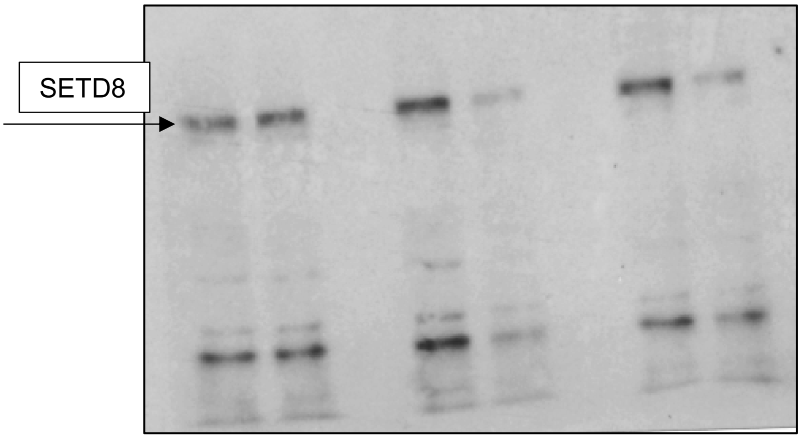

Full and uncropped western blot for Fig 5C
b-tubulin GB-1 (lanes 2-3) GB-2 (lanes 5-6) GB-3 (lanes 8-9)

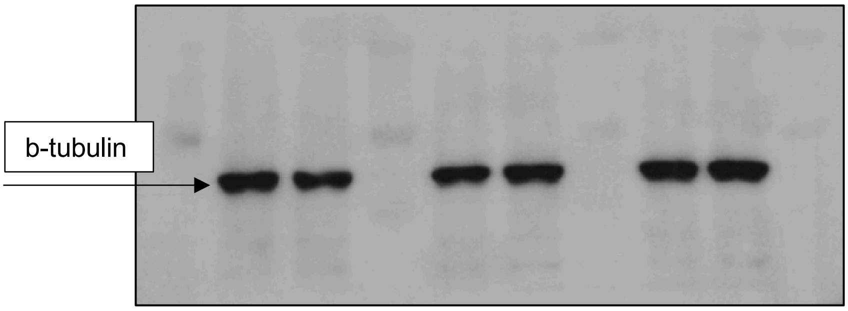

Full and uncropped western blot for Fig 5C
p21 GB-1 (lanes 1-2)

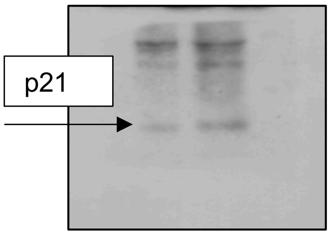

Full and uncropped western blot for Fig 5C
p21 GB-2 (lanes 1-2) GB-3 (4-5)

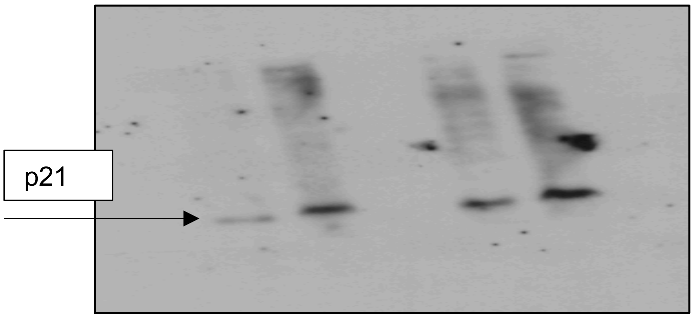

Supplement: Supplementary file 2 — Supplementary figures [file 41419_2023_6167_MOESM2_ESM.pdf]
