# Supplementary material for: Targeted inhibition of the methyltransferase SETD8 synergizes with the Wee1 inhibitor adavosertib in restraining glioblastoma growth
Source: Cell Death Dis. 2023 Sep 27;14(9):638. doi: 10.1038/s41419-023-06167-3 (PMC10533811; doi:10.1038/s41419-023-06167-3)
Supplement: Supplementary file 3 — Supplementary Materials and Methods [file 41419_2023_6167_MOESM3_ESM.pdf]

Glioblastoma primary cell isolation

The tumors were cut into small pieces and minced. The minced samples were prepared using the gentleMACS Dissociator in combination with the Tumor Dissociation Kit, human (Miltenyi Biotec, Cologne, Germany). The samples were then transferred into the gentleMACS C Tube. Once the C Tube was attached to the sleeve of gentleMACS Dissociator, different programs were run according to the protocol. The obtained cell suspension was then applied to a MACS SmartStrainer, mesh size 70 µm, placed on a 50 mL tube. The cell strainer was washed with 20 mL of RPMI 1640 and the cell suspension was centrifuged at $300 \times g$ for 5 min. The supernatant was completely aspirated, the cells resuspended and cultured.

Real-time PCR

RNA was extracted using RNeasy mini kit (Qiagen, Hilde, Germany) following the manufacturer's instructions. The integrity of the RNA was determined using NanoDrop 2000 (Thermo Fisher Scientific, St. Louis, MO, USA). Recombinant DNase (Qiagen) was used to remove potentially contaminating genomic DNA. RNA of each sample (1 µg of the extracted RNA) was firstly denatured and then reverse-transcribed using QuantiTect Reverse Transcription kit (Qiagen), following the manufacturer's instructions. Real-time PCR amplifications were performed using LightCycler 480 SYBR Green I Master (Roche Diagnostic, Rotkreuz, Switzerland) in a LightCycler480 Real Time thermocycler. The following protocol was adopted: 10 s for initial denaturation at 95 °C followed by 40 cycles consisting of 10 s at 94 °C for denaturation, 10 s at 60 °C for annealing, and 6 s for elongation at 72 °C temperature.

Time-lapse microscopy

LN-18 cells were pre-treated for 12h with adavosertib. Then, 4′,6-diamidino-2-phenylindole (DAPI; Sigma-Aldrich, St. Louis, MO, USA) and DMSO or UNC0379 were added. Cells were filmed with the Celldiscoverer 7 (Zeiss, Oberkochen, Germany) over 8h and pictures were taken every 1h.

Immunofluorescence

Cells were grown on microscopy slides, washed in PBS, fixed with 4% paraformaldehyde in PBS for 10 min and permeabilized with 0.2% Triton X-100 in PBS for further 10 min. For p-γ-H2AX staining, after permeabilization with Triton X-100, cells were incubated in ice cold methanol for 10 min. After blocking with 3% BSA in PBS for 1h, samples were incubated with primary antibodies in PBS + 1% BSA for 3h. After 3 PBS washes, samples were incubated in PBS + 1% BSA for 1h at room temperature with secondary antibodies.

Antibodies

The glioma array was immunostained with anti-SETD8 antibody (Cat. No. 14063-1-AP, ProteinTech, Rosemont, IL, USA).

Immunofluorescence was performed using anti-p-γ-H2AX (Cat. No. ab22551, Abcam, Cambridge, UK), anti-GFAP (Cat. No. sc-33673, Santa Cruz Biotechnology, Dallas, TX, USA) and anti-β-tubulin (Cat. No. sc-166729, Santa Cruz Biotechnology) antibodies. Secondary antibodies were purchased from Thermo Fisher Scientific: Alexa Fluor 568 goat-anti rabbit (Cat. No. A-11036) and goat-anti mouse (Cat. No. A-11029).

Immunoblots were incubated with primary antibodies recognizing p21 (Cat. No. ab109520, Abcam), p-S345 Chk1 (Cat. No ab58567, Abcam), Chk1 (Cat. No ab40866, Abcam), p53 (Cat. No. ab1101, Abcam), H4K20me1 (Cat. No. sc-134221, Santa Cruz Biotechnology), SETD8

(Cat. No. sc-377034, Santa Cruz Biotechnology), PARP (Cat. No. sc-8007, Santa Cruz Biotechnology), cleaved-caspase 3 (Cat. No. ab32042, Abcam), β-tubulin (Cat. No. sc-166729, Santa Cruz Biotechnology), β-actin (Cat. No. 13E5, Cell Signaling Technology, Danvers, MA, USA). Secondary antibodies were purchased from Cytiva (Marlborough, MA, USA): anti-rabbit HRP-linked (Cat. No. NA934) and anti-mouse HRP-linked (Cat. No. NA931).
